# Development of Prolonged Apnoea in a Suxamethonium-Naïve Patient During Electroconvulsive Therapy: A Case Report

**DOI:** 10.7759/cureus.72344

**Published:** 2024-10-25

**Authors:** Alican Bulut, Basavaraja Papanna, Bushra Ahmed, Kapil Kulkarni, Balamurugan Ramalingam

**Affiliations:** 1 Psychiatry, Essex Partnership University NHS Foundation Trust, Colchester, GBR; 2 Anesthesiology, East Suffolk and North Essex NHS Foundation Trust, Colchester, GBR

**Keywords:** anesthesia complications in ect, drug-related side effects and adverse reactions, pre-procedural assessment, pseudocholinesterase deficiency, suxamethonium-induced apnoea

## Abstract

Suxamethonium is commonly used as a muscle relaxant during electroconvulsive therapy (ECT). Prolonged apnoea associated with suxamethonium, mostly caused by pseudocholinesterase deficiency, is rare, but it can sometimes pose a great challenge in managing emergency situations due to patients’ inability to breathe; occasionally, it can result in serious complications, including death.

We report a case of a young male who had no prior exposure to suxamethonium and developed prolonged apnoea after induction with it. There was no record of any sensitivity or adverse reactions to any drugs for this patient or his family.

ECT is a well-known treatment for many psychiatric disorders when other treatments are ineffective. This case emphasizes the need for a comprehensive pre-procedural assessment, careful consideration, and the gathering of crucial information about potential sensitivities and adverse reactions associated with medications commonly used during the ECT procedure, especially when administering these medications for the first time.

## Introduction

Electroconvulsive therapy (ECT) is an effective treatment for several psychiatric disorders, including depression, bipolar disorder, catatonia, and schizophrenia [1-5]. Anaesthetic medications and muscle relaxants are essential parts of ECT to minimize potential risks such as fractures or self-injury [6,7].

Suxamethonium, also known as succinylcholine, is a commonly used muscle relaxant during ECT [8]. It has been extensively used in clinical practice since 1951 [9]. It is a short-acting muscle relaxant that produces muscle paralysis by attaching to the postsynaptic cholinergic receptors of the motor end plate [10].

Suxamethonium can sometimes lead to serious adverse effects, such as hyperkalaemia, malignant hyperthermia, and prolonged apnoea. Prolonged apnoea may occur due to pseudocholinesterase deficiency, old age, impaired liver function, impaired renal function, and medication interactions [10]. Sensitivity to suxamethonium is a well-known risk factor, and pre-procedural assessment for sensitivities is crucial in ECT safety protocols.

This case report presents the development of prolonged apnoea in a patient after induction with suxamethonium during their first ECT session, despite the absence of any known sensitivities and adverse reactions to any medication.

## Case presentation

The patient is a 22-year-old university student of North African descent currently living in the UK under a student visa, with no previous contact with mental health services. He was admitted to a psychiatric ward under section 2 of the MHA due to the gradual onset of bizarre behaviour, paranoid ideation, and a lack of insight into his condition. He had been homeless for a year and had no contact with family and friends during this period. He was detained when he went to Luton airport in a dishevelled and confused state, asking for flight tickets to the USA but having no money to do so. (He had a USA passport, which he misplaced while he was on the streets.) His family had filed a missing person’s complaint from Egypt in the UK, and his friends were unaware of his whereabouts.

In terms of clinical presentation on the ward, the patient was very difficult to engage in conversation, refused bloods, ECG, and medications, and would not attend ward reviews. His drinking and eating were poor, and he lost a significant amount of weight. There was evidence of self-neglect, mutism, maintaining postures for long hours, ambivalence, and ambitendency. He was hearing voices commanding him not to eat and drink, not to sit from a standing position, and to be awake all night. He had no insight into his mental health difficulties and would obey the voices due to extreme fear and anxiety. There was no history of alcohol or drug use while he was on the streets, and a CT scan had been done earlier in the general hospital to rule out any organic cause of his presentation. He was started on aripiprazole in the ward as he did not agree to bloods and ECG, the dose of which was further increased. He did not accept oral medications at times, and hence a depot aripiprazole injection was prescribed in case he refused oral medications. His mental state, however, deteriorated, and his BMs at one stage were less than 4 mmol/L as he refused to eat and drink, and he was sent to the general hospital. Due to no response to aripiprazole, he was switched from aripiprazole to olanzapine, and an urgent workup for ECT was planned. A second-opinion doctor assessed him, and he was given two ECT treatments under an emergency treatment order. During the switch from aripiprazole to olanzapine and when he received the first ECT treatment, he developed neck dystonia, which responded well to promethazine. However, he never developed any further side effects from the increased dose of olanzapine.

The patient underwent a total of 18 sessions of ECT with second opinion-appointed doctor authorization. However, during the induction of anaesthesia for the first session using propofol 110 mg and suxamethonium 25 mg, he experienced a significantly prolonged recovery period of approximately 40-45 minutes. A similar episode occurred during the second session as well. Subsequent investigation utilizing neuromuscular monitoring revealed an unexpected prolongation of muscle paralysis induced by suxamethonium. To ensure safety, he was maintained under anaesthesia and ventilated until complete recovery, for approximately 45 minutes. These findings were consistent with a variant pseudocholinesterase deficiency. To mitigate the risk of recurrence, the patient's subsequent ECT sessions were managed using an alternative muscle relaxant, rocuronium 30 mg, without encountering any further complications. A multi-disciplinary meeting was organized, involving anaesthesiologists, psychiatrists, ECT nurses, and the patient's family. The family was living abroad. His presentation and potential genetic basis for this reaction were explained to the family. It was emphasized to the family that genetic testing should be considered to check for pseudocholinesterase deficiency, as this can potentially cause a challenge for them as well when considering any intervention in the future. This presentation gave an opportunity to explore and discuss this condition with the patient and his family members.

The patient improved well following the ECT course, with significant positive engagement in conversations along with improvement in eating, drinking, and self-care.

## Discussion

Approximately one in 3,200 to one in 5,000 individuals have pseudocholinesterase deficiency, which is more prevalent in populations such as the Persian Jewish community and Alaska Natives [11]. There have been studies attempting genetic screening among the Persian Jewish community for various inherited conditions, including pseudocholinesterase deficiency [11]. This can be genetic as well as an acquired disorder that affects the body's ability to metabolize choline esters such as succinylcholine and mivacurium [11]. It can be caused by mutations in the BCHE gene [11]. This gene provides instructions for making the pseudocholinesterase enzyme, also known as butyrylcholinesterase, which is produced by the liver. Due to genetic causes, this condition is inherited in an autosomal recessive pattern [11].

Suxamethonium-induced prolonged apnoea is unpredictable and can be difficult to predict before ECT. There have been multiple case reports and various articles highlighting this risk and the seriousness of complications it can cause during the procedure [12-15]. Recent case reports published in 2018 showed that patients with suxamethonium-induced prolonged apnoea may need post-procedural ventilation for as long as 18 hours [16]. In the case report, the patient's age of 64 and history of previous prolonged apnoea likely contributed to the extended recovery period [16]. In contrast, our younger patient may have had a higher level of pseudocholinesterase activity, resulting in a shorter recovery time. These articles discussed how difficult it can be to screen for this phenomenon on initial assessment and also about the fact that this phenomenon has been forgotten over the years of clinical practice.

Articles published back in 1975 discussed this phenomenon and attempted to find a link between various diagnoses and their risk of pseudocholinesterase deficiency causing suxamethonium-induced prolonged apnoea [17]. They found that establishing an accurate diagnosis was difficult as there was a significant overlap of symptoms, and many of the patients were considered to have mixed symptoms of various diagnoses like schizophrenia, depression, and Huntington’s chorea [17]. It was interesting to note that Huntington’s chorea was seen in one out of 12 individuals having suxamethonium sensitivity [17].

When suxamethonium-induced prolonged apnoea is noticed in clinical practice, it's worth discussing this with the family and advising them about relevant testing to check for genetic predisposition, if any. This can be challenging as patients and their relatives often do not have any symptoms and find it hard to understand this condition. As a safe alternative, rocuronium has become a more viable option; this being a non-depolarizing agent makes pseudocholinesterase deficiency irrelevant [18].

## Conclusions

This case report confirms findings available from previous literature that suxamethonium-induced prolonged apnoea during ECT is a rare and unpredictable reaction and can potentially endanger the patient’s life. This case report also highlights the need for thorough assessment and information gathering before starting therapy. If patients are at risk of developing suxamethonium-induced prolonged apnoea or develop it during treatment, this warrants the use of alternative medication and genetic counselling of family members.
